# Supplementary material for: Audit and feedback to change diagnostic image ordering practices: A systematic review and meta-analysis
Source: PLoS One. 2024 Jun 5;19(6):e0300001. doi: 10.1371/journal.pone.0300001 (PMC11152319; doi:10.1371/journal.pone.0300001)
Supplement: S1 Appendix — S1 Fig. a. Effect of audit and feedback in observational studies on the number of diagnostic imaging requests (continuous outcome) (4–6). b. Effect of audit and feedback in observational studies on the number of diagnostic imaging requests (dichotomous outcome) (7, 8). S2 Fig. Effect of audit and feedback in observational studies on image order appropriateness (dichotomous outcome) (7). S3 Fig. Funnel plot of RCTs analyzing the total image order outcome. We did not consider this figure to be indicative of publication bias. The study in the bottom right favored the control intervention, not AF. S4 Fig. Funnel plot of RCTS analyzing the appropriateness of image orders outcome.We did not consider this figure to be indicative of publication bias. S1 Table. Description of AF interventions using TiDIER recommendations (1). Abbreviations: AF, Audit and Feedback; CT, Computed Tomography; Echo, Echocardiography; GIM, General physicians; Res, residents; Gov., Government; Mm; MRI, Magnetic Resonance Imaging; N/A, not applicable; PCP, Primary care physicians (e) PCPs refers to primary care physicians and may include family, general practice and general internal medicine physicians, (f) The term residents also refers to registrars (g) Comparison provided Includes own/ peers’ previous performance, national benchmark. Note: For multifaceted interventions, we assessed the characteristics of the audit and feedback component. S2 Table. a. Risk of Bias for NRCTs using the Risk Of Bias In Non-randomized Studies—of Interventions (ROBINS-I) tool (2). b. Risk of Bias for observational studies using Effective Practice and Organisation of Care (EPOC) recommendations (3). c. Risk of Bias for interrupted time series studies using Effective Practice and Organisation of Care (EPOC) recommendations (3). Legend: ● Low risk; ● Indeterminate Risk; ● High risk. S3 Table. Effect of audit and feedback in a non-randomized, crossover design study on the number of diagnostic imaging request 9).*no p-valu [file pone.0300001.s001.zip › S1_File.docx]

| #15 | #3 AND #6 AND #9 AND #12 AND [article] /lim AND [english]/lim AND [embase]/lim AND [humans]/lim |
| --- | --- |
| #14 | #3 AND #6 AND #9 AND #12 AND [article]/lim AND [english]/lim AND [embase]/lim |
| #13 | #3 AND #6 AND #9 AND #12 |
| #12 | #10 AND #11 |
| #11 | 'health personnel':ti,ab OR 'healthcare personnel':ti,ab OR 'health care personnel':ti,ab OR 'family medicine':ti,ab OR 'family practice':ti,ab OR 'family practitioner':ti,ab OR 'general practice':ti,ab OR 'general practitioner':ti,ab OR 'primary care':ti,ab |
| #10 | 'general practice':ti,ab OR 'primary health care':ti,ab OR 'general practitioner':ti,ab OR 'family nurse practitioner':ti,ab |
| #9 | #7 OR #8 |
| #8 | 'practice pattern':ti,ab OR 'practice patterns':ti,ab OR 'quality assurance':ti,ab OR 'quality indicators':ti,ab OR 'ordering behaviour':ti,ab OR 'ordering behavior':ti,ab OR 'behaviour change':ti,ab OR 'behavior change':ti,ab OR 'ordering tests':ti,ab |
| #7 | 'health care quality'/exp OR 'patient referral'/exp OR 'consultation'/exp OR 'unnecessary procedure'/exp OR 'quality control'/exp OR 'outcome assessment'/exp |
| #6 | #4 OR #5 |
| #5 | ('diagnostic imaging':ti,ab OR radiograph*:ti,ab OR radiogram*:ti,ab OR radiolog*:ti,ab OR xray:ti,ab OR 'x ray':ti,ab OR mri:ti,ab OR 'magnetic resonance imaging' OR ultrasound:ti,ab OR ultrasonography) AND ti, AND ab OR 'computed tomography':ti,ab |
| #4 | 'clinical laboratory'/exp OR 'diagnostic test'/exp OR 'diagnostic imaging'/exp OR 'laboratory technique'/exp OR 'radiology department'/exp |
| #3 | #1 OR #2 |
| #2 | audit*:ti,ab OR feedback:ti,ab OR benchmark*:ti,ab OR 'chart review':ti,ab OR 'chart reviews':ti,ab |
| #1 | 'clinical audit'/exp OR 'nursing audit'/exp OR 'benchmarking'/exp OR 'health care utilization'/exp OR 'utilization review'/exp |
