# Supplementary material for: Audit and feedback to change diagnostic image ordering practices: A systematic review and meta-analysis
Source: PLoS One. 2024 Jun 5;19(6):e0300001. doi: 10.1371/journal.pone.0300001 (PMC11152319; doi:10.1371/journal.pone.0300001)
Supplement: S1 Appendix — S1 Fig. a. Effect of audit and feedback in observational studies on the number of diagnostic imaging requests (continuous outcome) (4–6). b. Effect of audit and feedback in observational studies on the number of diagnostic imaging requests (dichotomous outcome) (7, 8). S2 Fig. Effect of audit and feedback in observational studies on image order appropriateness (dichotomous outcome) (7). S3 Fig. Funnel plot of RCTs analyzing the total image order outcome. We did not consider this figure to be indicative of publication bias. The study in the bottom right favored the control intervention, not AF. S4 Fig. Funnel plot of RCTS analyzing the appropriateness of image orders outcome.We did not consider this figure to be indicative of publication bias. S1 Table. Description of AF interventions using TiDIER recommendations (1). Abbreviations: AF, Audit and Feedback; CT, Computed Tomography; Echo, Echocardiography; GIM, General physicians; Res, residents; Gov., Government; Mm; MRI, Magnetic Resonance Imaging; N/A, not applicable; PCP, Primary care physicians (e) PCPs refers to primary care physicians and may include family, general practice and general internal medicine physicians, (f) The term residents also refers to registrars (g) Comparison provided Includes own/ peers’ previous performance, national benchmark. Note: For multifaceted interventions, we assessed the characteristics of the audit and feedback component. S2 Table. a. Risk of Bias for NRCTs using the Risk Of Bias In Non-randomized Studies—of Interventions (ROBINS-I) tool (2). b. Risk of Bias for observational studies using Effective Practice and Organisation of Care (EPOC) recommendations (3). c. Risk of Bias for interrupted time series studies using Effective Practice and Organisation of Care (EPOC) recommendations (3). Legend: ● Low risk; ● Indeterminate Risk; ● High risk. S3 Table. Effect of audit and feedback in a non-randomized, crossover design study on the number of diagnostic imaging request 9).*no p-valu [file pone.0300001.s001.zip › S1_Table.docx]

|  | **Berwick et al., 1986** | **Freeborn et al., 1997** | **Cammisa et al., 2011** | **Bhatia et al., 2013** | **Morgan et al., 2019** | **Halpern et al., 2020** |
| --- | --- | --- | --- | --- | --- | --- |
| **Who and where?** |  |  |  |  |  |  |
| Provider type | PCPs (a) | PCPs | PCPs | GIM teams | PCPs, PCP Res.(b) | PCPs |
| AF provided to Individuals or group | Individual | Individual | Individual | Group | Individual | Individual |
| AF delivered directly to provider? | Yes | Yes | Yes | Yes | Yes | Yes |
| Inpatient/Outpatient setting? | Outpatient | Outpatient | Outpatient | Inpatient | Outpatient | Outpatient |
| **Content of AF reports** |  |  |  |  |  |  |
| Imaging modality | X-Ray | X-Ray, CT, MRI | MRI | Echo | X-Ray, CT | Multiple |
| Desired change in ordering | Decrease | Decrease | Decrease | Decrease | Decrease | Decrease |
| Patient outcomes (findings on imaging test) | No | No | No | Yes | No | No |
| Other info (e.g. costs, guidelines, doses) | Yes | No | Yes | Yes | Yes | No |
| AF of Individual provider or group | Individual | Individual | Individual | Group | Individual | Individual |
| Feedback about Individual cases or aggregate cases | Aggregate | Aggregate | Aggregate | Unclear | Aggregate | Aggregate |
| Comparison provided (c) | Peers | Own, Peers | Peers | None | Peers | Own, Peers |
| Graphical elements | No | Yes | Yes | No | No | No |
| **When and how much AF?** |  |  |  |  |  |  |
| Time period of audit data | 1 Month | 2-3 Months | 12 Months | 2 Weeks | Unclear | 2 Years |
| Lag between audit and feedback | Brief | Months | Unclear | Brief | Unclear | Weeks |
| Frequency (number of times given) | 3 | 3 | Unclear | 2 | 1 | 12 |
| Time in between reports | 8 Weeks | 2 Months | Unclear | 2 Weeks | N/A | 1 Month |
| Duration of intervention (months) | 12 | 6 | 12 | 4 | 20 | 12 |
| **How was AF delivered?** |  |  |  |  |  |  |
| Verbal or written | Written | Written | Both | Written | Written | Written |
| Delivery mode | Unclear | Post | In Person | E-mail | Post | E-mail, Post |
| Source of IP delivery | N/A | N/A | Led | N/A | N/A | N/A |
| Asked to reflect on AF | Yes | No | Yes | No | Yes | No |
| **Who provided AF?** |  |  |  |  |  |  |
| Who conducted the audit? | Researcher | Both | Company | Researcher | Gov. | Researcher |
| Who developed the feedback report? | Researcher | Both | Company | Researcher | Researcher | Researcher |
| **Fidelity** |  |  |  |  |  |  |
| Planned assessment of AF receipt? | Not Reported | Yes | Not Reported | Not Reported | Not Reported | Not Reported |
| AF receipt by providers | Not Reported | Not Reported | Not Reported | Not Reported | Not Reported | Not Reported |
