# Supplementary material for: Audit and feedback to change diagnostic image ordering practices: A systematic review and meta-analysis
Source: PLoS One. 2024 Jun 5;19(6):e0300001. doi: 10.1371/journal.pone.0300001 (PMC11152319; doi:10.1371/journal.pone.0300001)
Supplement: S1 Appendix — S1 Fig. a. Effect of audit and feedback in observational studies on the number of diagnostic imaging requests (continuous outcome) (4–6). b. Effect of audit and feedback in observational studies on the number of diagnostic imaging requests (dichotomous outcome) (7, 8). S2 Fig. Effect of audit and feedback in observational studies on image order appropriateness (dichotomous outcome) (7). S3 Fig. Funnel plot of RCTs analyzing the total image order outcome. We did not consider this figure to be indicative of publication bias. The study in the bottom right favored the control intervention, not AF. S4 Fig. Funnel plot of RCTS analyzing the appropriateness of image orders outcome.We did not consider this figure to be indicative of publication bias. S1 Table. Description of AF interventions using TiDIER recommendations (1). Abbreviations: AF, Audit and Feedback; CT, Computed Tomography; Echo, Echocardiography; GIM, General physicians; Res, residents; Gov., Government; Mm; MRI, Magnetic Resonance Imaging; N/A, not applicable; PCP, Primary care physicians (e) PCPs refers to primary care physicians and may include family, general practice and general internal medicine physicians, (f) The term residents also refers to registrars (g) Comparison provided Includes own/ peers’ previous performance, national benchmark. Note: For multifaceted interventions, we assessed the characteristics of the audit and feedback component. S2 Table. a. Risk of Bias for NRCTs using the Risk Of Bias In Non-randomized Studies—of Interventions (ROBINS-I) tool (2). b. Risk of Bias for observational studies using Effective Practice and Organisation of Care (EPOC) recommendations (3). c. Risk of Bias for interrupted time series studies using Effective Practice and Organisation of Care (EPOC) recommendations (3). Legend: ● Low risk; ● Indeterminate Risk; ● High risk. S3 Table. Effect of audit and feedback in a non-randomized, crossover design study on the number of diagnostic imaging request 9).*no p-valu [file pone.0300001.s001.zip › S4_File.docx]

References for supporting information

1. Hoffmann TC, Glasziou PP, Boutron I, Milne R, Perera R, Moher D, et al. Better reporting of interventions: template for intervention description and replication (TIDieR) checklist and guide. BMJ : British Medical Journal. 2014;348:g1687. doi: 10.1136/bmj.g1687.

2. Sterne JA, Hernán MA, Reeves BC, Savović J, Berkman ND, Viswanathan M, et al. ROBINS-I: a tool for assessing risk of bias in non-randomised studies of interventions. BMJ. 2016;355:i4919. doi: 10.1136/bmj.i4919.

3. Cochrane Effective Practice and Organisation of Care Working Group. EPOC resources for review authors Oslo, Norway: Norwegian Institute of Public Health; 2021 [updated January 2022]. Available from: <https://epoc.cochrane.org/resources/epoc-resources-review-authors>.

4. Freeborn DK, Shye D, Mullooly JP, Eraker S, Romeo J. Primary care physicians' use of lumbar spine imaging tests: effects of guidelines and practice pattern feedback. 1997;12(10):619-25. doi: 10.1046/j.1525-1497.1997.07122.x. PubMed PMID: 9346458.

5. Zafar HM, Ip IK, Mills AM, Raja AS, Langlotz CP, Khorasani R. Effect of Clinical Decision Support-Generated Report Cards Versus Real-Time Alerts on Primary Care Provider Guideline Adherence for Low Back Pain Outpatient Lumbar Spine MRI Orders. AJR Am J Roentgenol. 2019;212(2):386-94. doi: 10.2214/AJR.18.19780. PubMed PMID: 30476451.

6. Cammisa C, Partridge G, Ardans C, Buehrer K, Chapman B, Beckman H. Engaging physicians in change: results of a safety net quality improvement program to reduce overuse. Am J Med Qual. 2011;26(1):26-33. Epub 20100927. doi: 10.1177/1062860610373380. PubMed PMID: 20876341; PubMed Central PMCID: PMCPMC3939773.

7. Bhatia RS, Milford CE, Picard MH, Weiner RB. An educational intervention reduces the rate of inappropriate echocardiograms on an inpatient medical service. JACC Cardiovasc Imaging. 2013;6(5):545-55. doi: 10.1016/j.jcmg.2013.01.010. PubMed PMID: 23582360.

8. Halpern DJ, Clark-Randall A, Woodall J, Anderson J, Shah K. Reducing Imaging Utilization in Primary Care Through Implementation of a Peer Comparison Dashboard. Journal of General Internal Medicine. 2020;36(1):108-13. doi: 10.1007/s11606-020-06164-8.

9. Berwick DM, Coltin KL. Feedback reduces test use in a health maintenance organization. Jama. 1986;255(11):1450-4. PubMed PMID: 3951079.

10. Morgan T, Wu J, Ovchinikova L, Lindner R, Blogg S, Moorin R. A national intervention to reduce imaging for low back pain by general practitioners: a retrospective economic program evaluation using Medicare Benefits Schedule data. BMC Health Serv Res. 2019;19(1):983. doi: 10.1186/s12913-019-4773-y.
