# Supplementary figures and images for: Audit and feedback to change diagnostic image ordering practices: A systematic review and meta-analysis
Source: PLoS One. 2024 Jun 5;19(6):e0300001. doi: 10.1371/journal.pone.0300001 (PMC11152319; doi:10.1371/journal.pone.0300001)

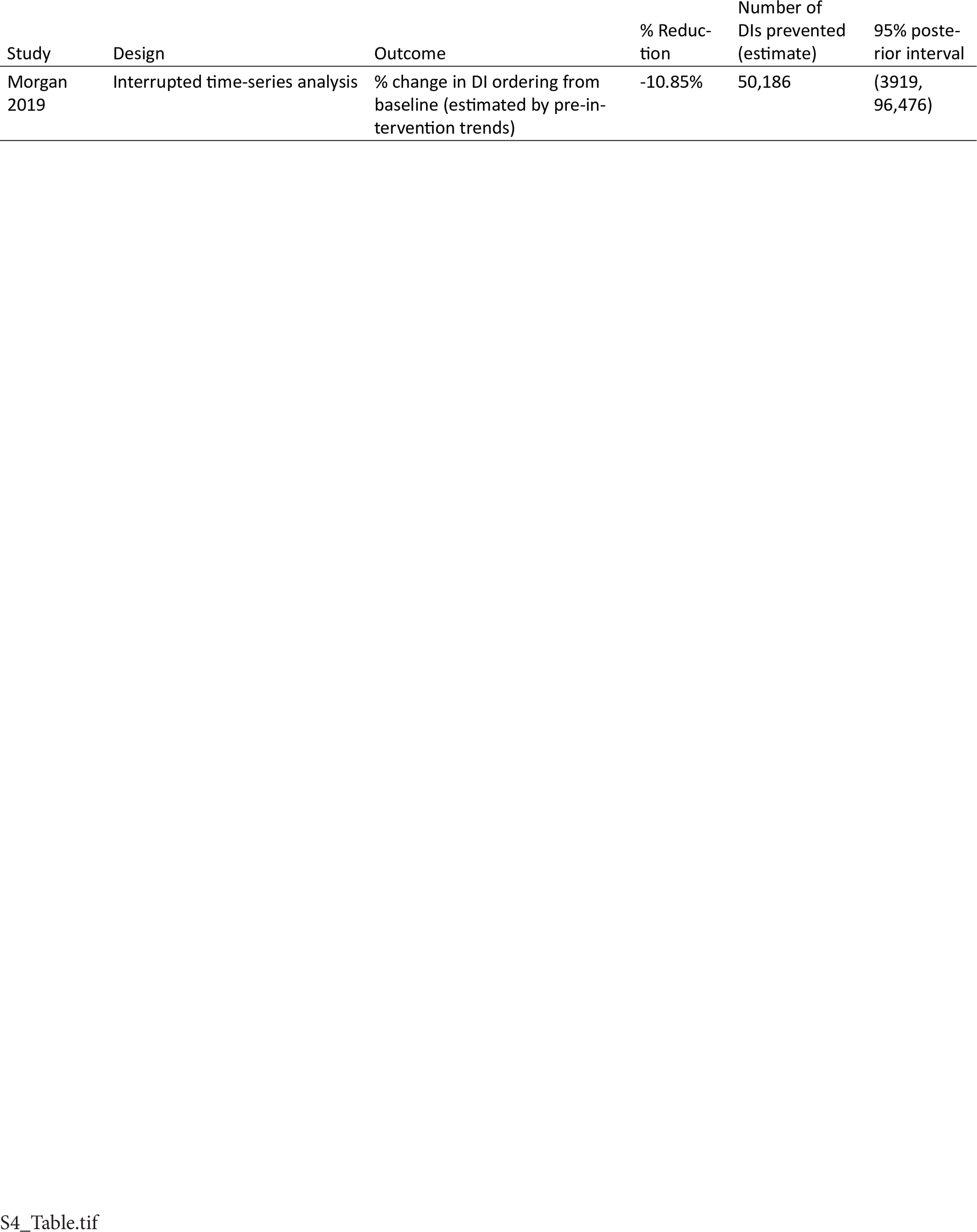

Supplement: S1 Appendix — S1 Fig. a. Effect of audit and feedback in observational studies on the number of diagnostic imaging requests (continuous outcome) (4–6). b. Effect of audit and feedback in observational studies on the number of diagnostic imaging requests (dichotomous outcome) (7, 8). S2 Fig. Effect of audit and feedback in observational studies on image order appropriateness (dichotomous outcome) (7). S3 Fig. Funnel plot of RCTs analyzing the total image order outcome. We did not consider this figure to be indicative of publication bias. The study in the bottom right favored the control intervention, not AF. S4 Fig. Funnel plot of RCTS analyzing the appropriateness of image orders outcome.We did not consider this figure to be indicative of publication bias. S1 Table. Description of AF interventions using TiDIER recommendations (1). Abbreviations: AF, Audit and Feedback; CT, Computed Tomography; Echo, Echocardiography; GIM, General physicians; Res, residents; Gov., Government; Mm; MRI, Magnetic Resonance Imaging; N/A, not applicable; PCP, Primary care physicians (e) PCPs refers to primary care physicians and may include family, general practice and general internal medicine physicians, (f) The term residents also refers to registrars (g) Comparison provided Includes own/ peers’ previous performance, national benchmark. Note: For multifaceted interventions, we assessed the characteristics of the audit and feedback component. S2 Table. a. Risk of Bias for NRCTs using the Risk Of Bias In Non-randomized Studies—of Interventions (ROBINS-I) tool (2). b. Risk of Bias for observational studies using Effective Practice and Organisation of Care (EPOC) recommendations (3). c. Risk of Bias for interrupted time series studies using Effective Practice and Organisation of Care (EPOC) recommendations (3). Legend: ● Low risk; ● Indeterminate Risk; ● High risk. S3 Table. Effect of audit and feedback in a non-randomized, crossover design study on the number of diagnostic imaging request 9).*no p-valu [file pone.0300001.s001.zip › S4_Table.tif]

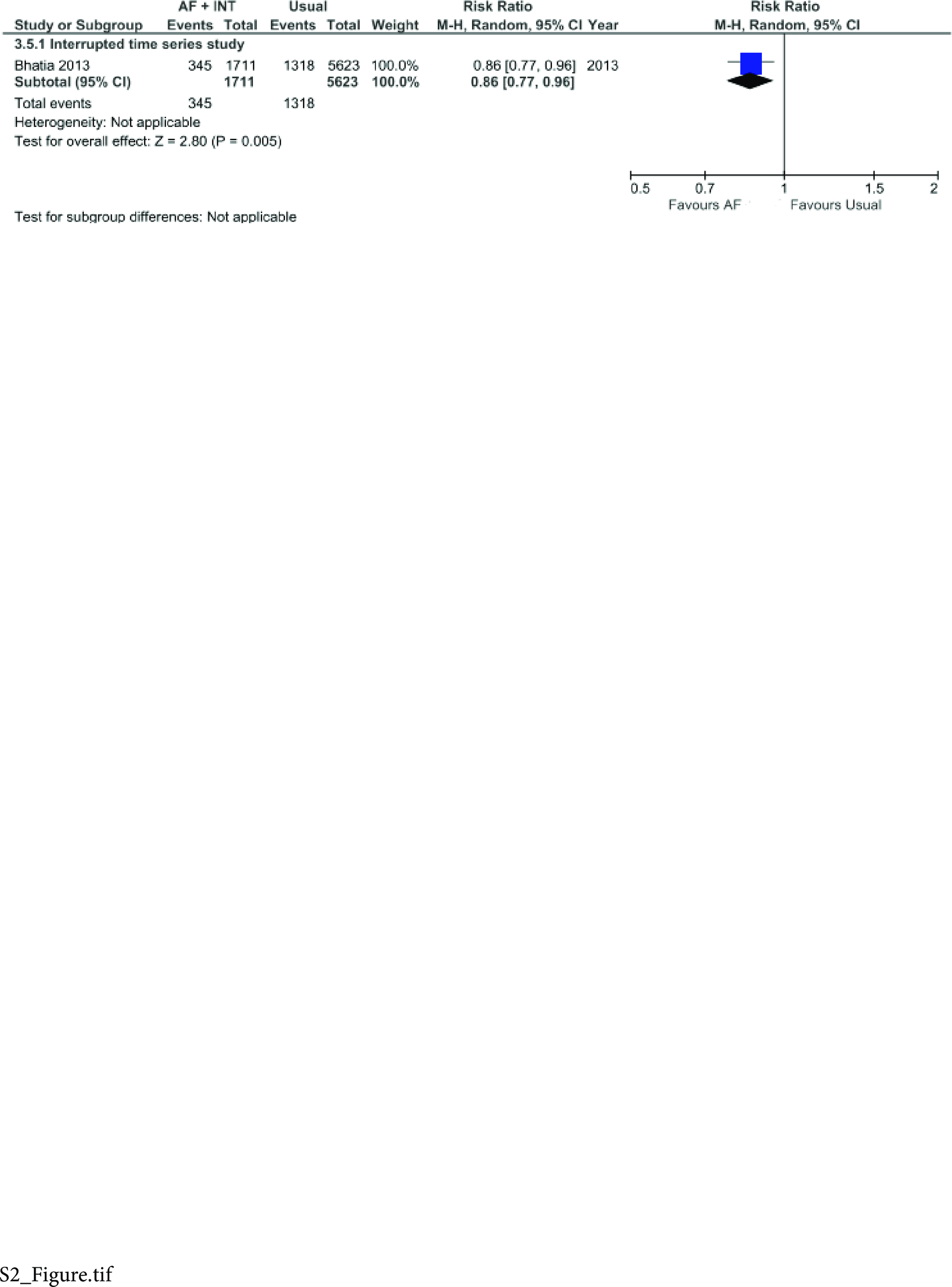

Supplement: S1 Appendix — S1 Fig. a. Effect of audit and feedback in observational studies on the number of diagnostic imaging requests (continuous outcome) (4–6). b. Effect of audit and feedback in observational studies on the number of diagnostic imaging requests (dichotomous outcome) (7, 8). S2 Fig. Effect of audit and feedback in observational studies on image order appropriateness (dichotomous outcome) (7). S3 Fig. Funnel plot of RCTs analyzing the total image order outcome. We did not consider this figure to be indicative of publication bias. The study in the bottom right favored the control intervention, not AF. S4 Fig. Funnel plot of RCTS analyzing the appropriateness of image orders outcome.We did not consider this figure to be indicative of publication bias. S1 Table. Description of AF interventions using TiDIER recommendations (1). Abbreviations: AF, Audit and Feedback; CT, Computed Tomography; Echo, Echocardiography; GIM, General physicians; Res, residents; Gov., Government; Mm; MRI, Magnetic Resonance Imaging; N/A, not applicable; PCP, Primary care physicians (e) PCPs refers to primary care physicians and may include family, general practice and general internal medicine physicians, (f) The term residents also refers to registrars (g) Comparison provided Includes own/ peers’ previous performance, national benchmark. Note: For multifaceted interventions, we assessed the characteristics of the audit and feedback component. S2 Table. a. Risk of Bias for NRCTs using the Risk Of Bias In Non-randomized Studies—of Interventions (ROBINS-I) tool (2). b. Risk of Bias for observational studies using Effective Practice and Organisation of Care (EPOC) recommendations (3). c. Risk of Bias for interrupted time series studies using Effective Practice and Organisation of Care (EPOC) recommendations (3). Legend: ● Low risk; ● Indeterminate Risk; ● High risk. S3 Table. Effect of audit and feedback in a non-randomized, crossover design study on the number of diagnostic imaging request 9).*no p-valu [file pone.0300001.s001.zip › S2_Fig.tif]

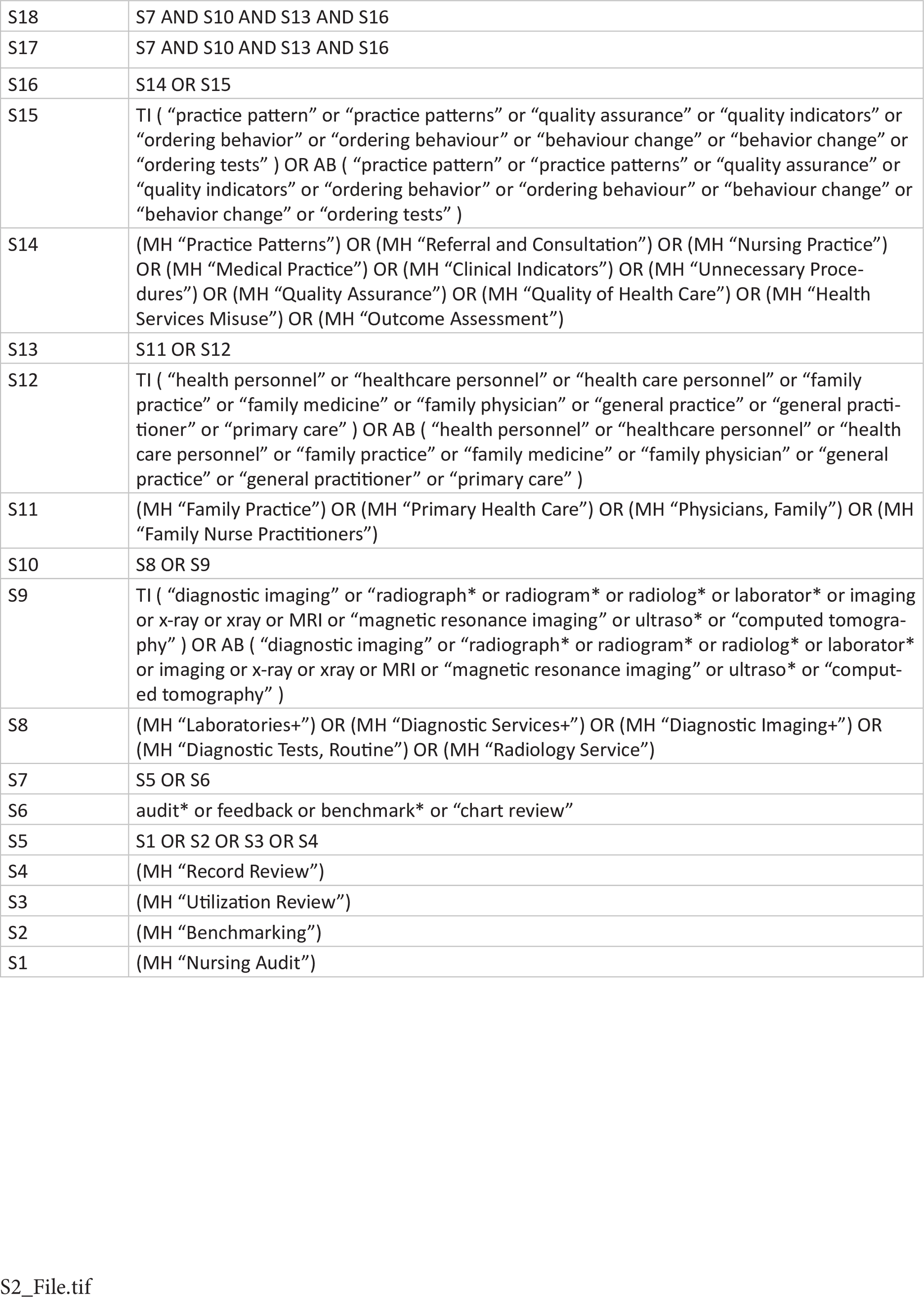

Supplement: S1 Appendix — S1 Fig. a. Effect of audit and feedback in observational studies on the number of diagnostic imaging requests (continuous outcome) (4–6). b. Effect of audit and feedback in observational studies on the number of diagnostic imaging requests (dichotomous outcome) (7, 8). S2 Fig. Effect of audit and feedback in observational studies on image order appropriateness (dichotomous outcome) (7). S3 Fig. Funnel plot of RCTs analyzing the total image order outcome. We did not consider this figure to be indicative of publication bias. The study in the bottom right favored the control intervention, not AF. S4 Fig. Funnel plot of RCTS analyzing the appropriateness of image orders outcome.We did not consider this figure to be indicative of publication bias. S1 Table. Description of AF interventions using TiDIER recommendations (1). Abbreviations: AF, Audit and Feedback; CT, Computed Tomography; Echo, Echocardiography; GIM, General physicians; Res, residents; Gov., Government; Mm; MRI, Magnetic Resonance Imaging; N/A, not applicable; PCP, Primary care physicians (e) PCPs refers to primary care physicians and may include family, general practice and general internal medicine physicians, (f) The term residents also refers to registrars (g) Comparison provided Includes own/ peers’ previous performance, national benchmark. Note: For multifaceted interventions, we assessed the characteristics of the audit and feedback component. S2 Table. a. Risk of Bias for NRCTs using the Risk Of Bias In Non-randomized Studies—of Interventions (ROBINS-I) tool (2). b. Risk of Bias for observational studies using Effective Practice and Organisation of Care (EPOC) recommendations (3). c. Risk of Bias for interrupted time series studies using Effective Practice and Organisation of Care (EPOC) recommendations (3). Legend: ● Low risk; ● Indeterminate Risk; ● High risk. S3 Table. Effect of audit and feedback in a non-randomized, crossover design study on the number of diagnostic imaging request 9).*no p-valu [file pone.0300001.s001.zip › S2_File.tif]

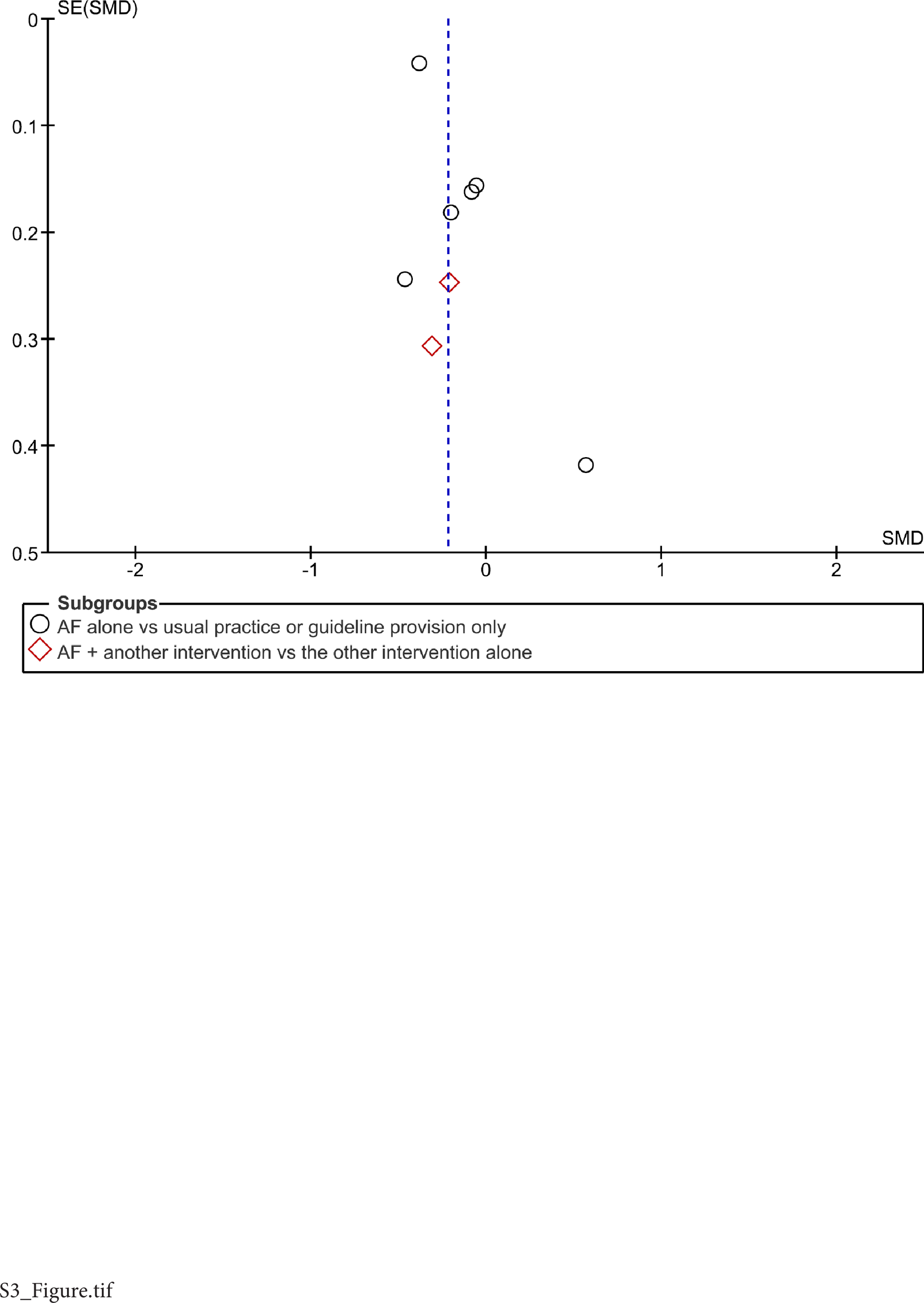

Supplement: S1 Appendix — S1 Fig. a. Effect of audit and feedback in observational studies on the number of diagnostic imaging requests (continuous outcome) (4–6). b. Effect of audit and feedback in observational studies on the number of diagnostic imaging requests (dichotomous outcome) (7, 8). S2 Fig. Effect of audit and feedback in observational studies on image order appropriateness (dichotomous outcome) (7). S3 Fig. Funnel plot of RCTs analyzing the total image order outcome. We did not consider this figure to be indicative of publication bias. The study in the bottom right favored the control intervention, not AF. S4 Fig. Funnel plot of RCTS analyzing the appropriateness of image orders outcome.We did not consider this figure to be indicative of publication bias. S1 Table. Description of AF interventions using TiDIER recommendations (1). Abbreviations: AF, Audit and Feedback; CT, Computed Tomography; Echo, Echocardiography; GIM, General physicians; Res, residents; Gov., Government; Mm; MRI, Magnetic Resonance Imaging; N/A, not applicable; PCP, Primary care physicians (e) PCPs refers to primary care physicians and may include family, general practice and general internal medicine physicians, (f) The term residents also refers to registrars (g) Comparison provided Includes own/ peers’ previous performance, national benchmark. Note: For multifaceted interventions, we assessed the characteristics of the audit and feedback component. S2 Table. a. Risk of Bias for NRCTs using the Risk Of Bias In Non-randomized Studies—of Interventions (ROBINS-I) tool (2). b. Risk of Bias for observational studies using Effective Practice and Organisation of Care (EPOC) recommendations (3). c. Risk of Bias for interrupted time series studies using Effective Practice and Organisation of Care (EPOC) recommendations (3). Legend: ● Low risk; ● Indeterminate Risk; ● High risk. S3 Table. Effect of audit and feedback in a non-randomized, crossover design study on the number of diagnostic imaging request 9).*no p-valu [file pone.0300001.s001.zip › S3_Fig.tif]

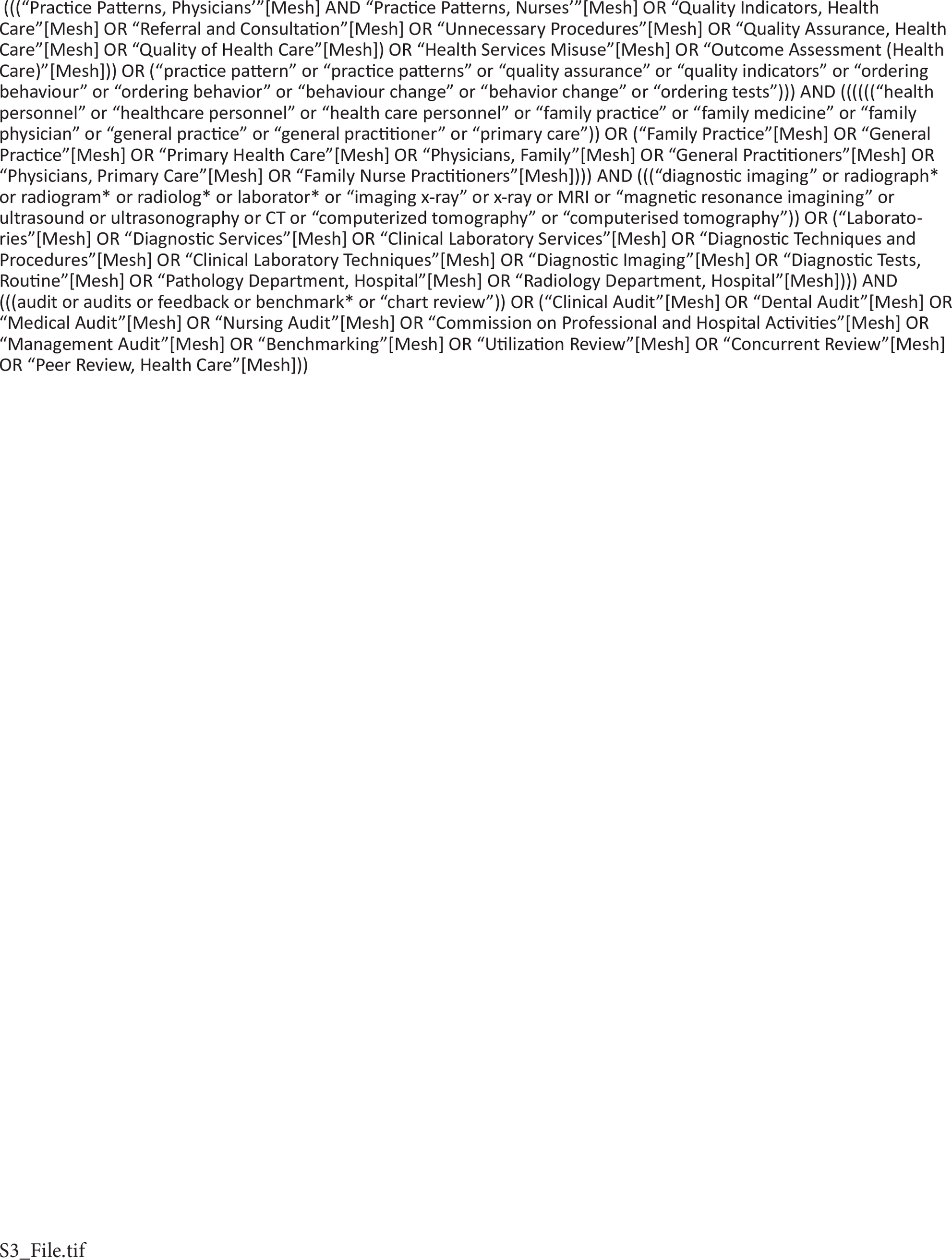

Supplement: S1 Appendix — S1 Fig. a. Effect of audit and feedback in observational studies on the number of diagnostic imaging requests (continuous outcome) (4–6). b. Effect of audit and feedback in observational studies on the number of diagnostic imaging requests (dichotomous outcome) (7, 8). S2 Fig. Effect of audit and feedback in observational studies on image order appropriateness (dichotomous outcome) (7). S3 Fig. Funnel plot of RCTs analyzing the total image order outcome. We did not consider this figure to be indicative of publication bias. The study in the bottom right favored the control intervention, not AF. S4 Fig. Funnel plot of RCTS analyzing the appropriateness of image orders outcome.We did not consider this figure to be indicative of publication bias. S1 Table. Description of AF interventions using TiDIER recommendations (1). Abbreviations: AF, Audit and Feedback; CT, Computed Tomography; Echo, Echocardiography; GIM, General physicians; Res, residents; Gov., Government; Mm; MRI, Magnetic Resonance Imaging; N/A, not applicable; PCP, Primary care physicians (e) PCPs refers to primary care physicians and may include family, general practice and general internal medicine physicians, (f) The term residents also refers to registrars (g) Comparison provided Includes own/ peers’ previous performance, national benchmark. Note: For multifaceted interventions, we assessed the characteristics of the audit and feedback component. S2 Table. a. Risk of Bias for NRCTs using the Risk Of Bias In Non-randomized Studies—of Interventions (ROBINS-I) tool (2). b. Risk of Bias for observational studies using Effective Practice and Organisation of Care (EPOC) recommendations (3). c. Risk of Bias for interrupted time series studies using Effective Practice and Organisation of Care (EPOC) recommendations (3). Legend: ● Low risk; ● Indeterminate Risk; ● High risk. S3 Table. Effect of audit and feedback in a non-randomized, crossover design study on the number of diagnostic imaging request 9).*no p-valu [file pone.0300001.s001.zip › S3_File.tif]

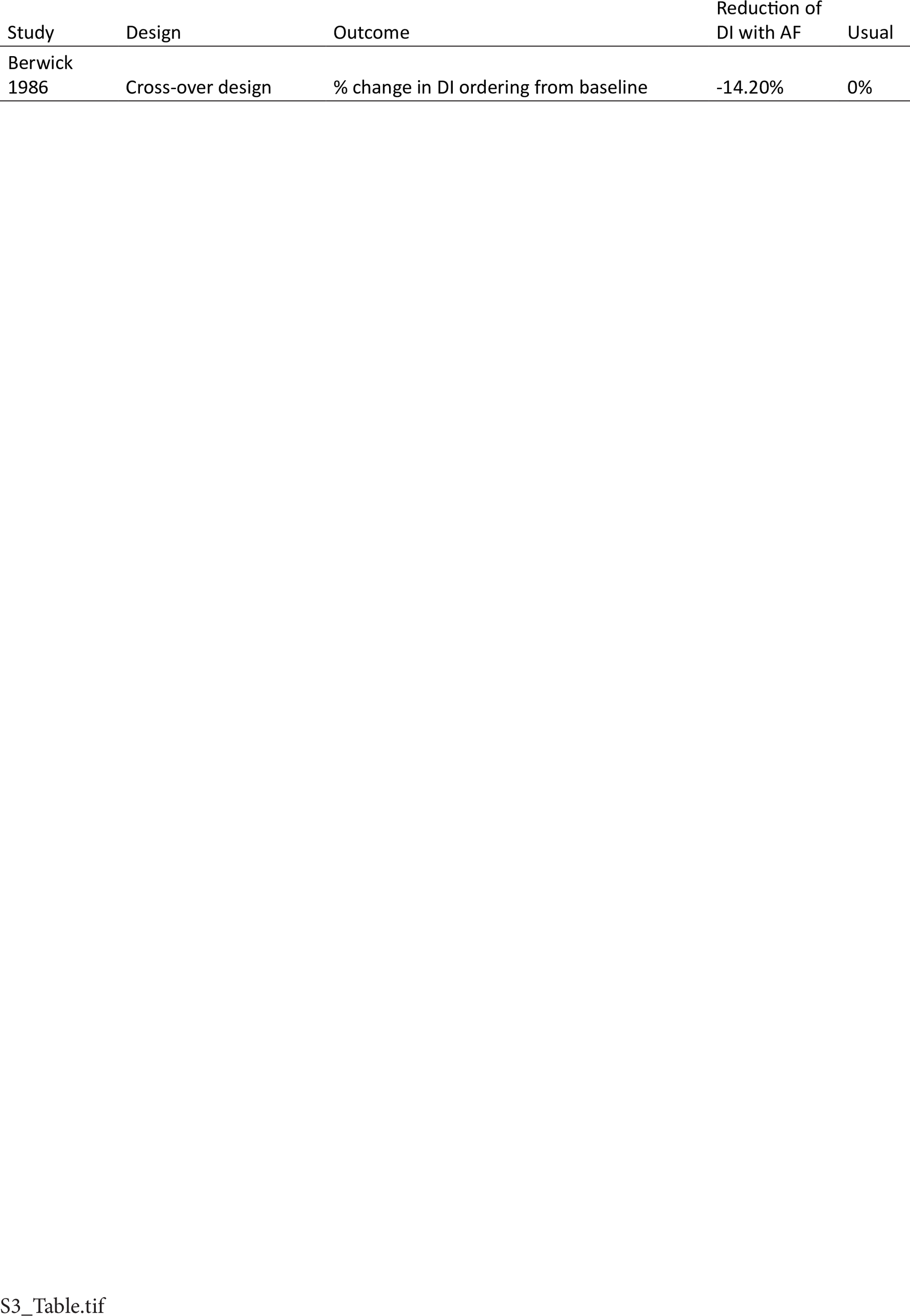

Supplement: S1 Appendix — S1 Fig. a. Effect of audit and feedback in observational studies on the number of diagnostic imaging requests (continuous outcome) (4–6). b. Effect of audit and feedback in observational studies on the number of diagnostic imaging requests (dichotomous outcome) (7, 8). S2 Fig. Effect of audit and feedback in observational studies on image order appropriateness (dichotomous outcome) (7). S3 Fig. Funnel plot of RCTs analyzing the total image order outcome. We did not consider this figure to be indicative of publication bias. The study in the bottom right favored the control intervention, not AF. S4 Fig. Funnel plot of RCTS analyzing the appropriateness of image orders outcome.We did not consider this figure to be indicative of publication bias. S1 Table. Description of AF interventions using TiDIER recommendations (1). Abbreviations: AF, Audit and Feedback; CT, Computed Tomography; Echo, Echocardiography; GIM, General physicians; Res, residents; Gov., Government; Mm; MRI, Magnetic Resonance Imaging; N/A, not applicable; PCP, Primary care physicians (e) PCPs refers to primary care physicians and may include family, general practice and general internal medicine physicians, (f) The term residents also refers to registrars (g) Comparison provided Includes own/ peers’ previous performance, national benchmark. Note: For multifaceted interventions, we assessed the characteristics of the audit and feedback component. S2 Table. a. Risk of Bias for NRCTs using the Risk Of Bias In Non-randomized Studies—of Interventions (ROBINS-I) tool (2). b. Risk of Bias for observational studies using Effective Practice and Organisation of Care (EPOC) recommendations (3). c. Risk of Bias for interrupted time series studies using Effective Practice and Organisation of Care (EPOC) recommendations (3). Legend: ● Low risk; ● Indeterminate Risk; ● High risk. S3 Table. Effect of audit and feedback in a non-randomized, crossover design study on the number of diagnostic imaging request 9).*no p-valu [file pone.0300001.s001.zip › S3_Table.tif]

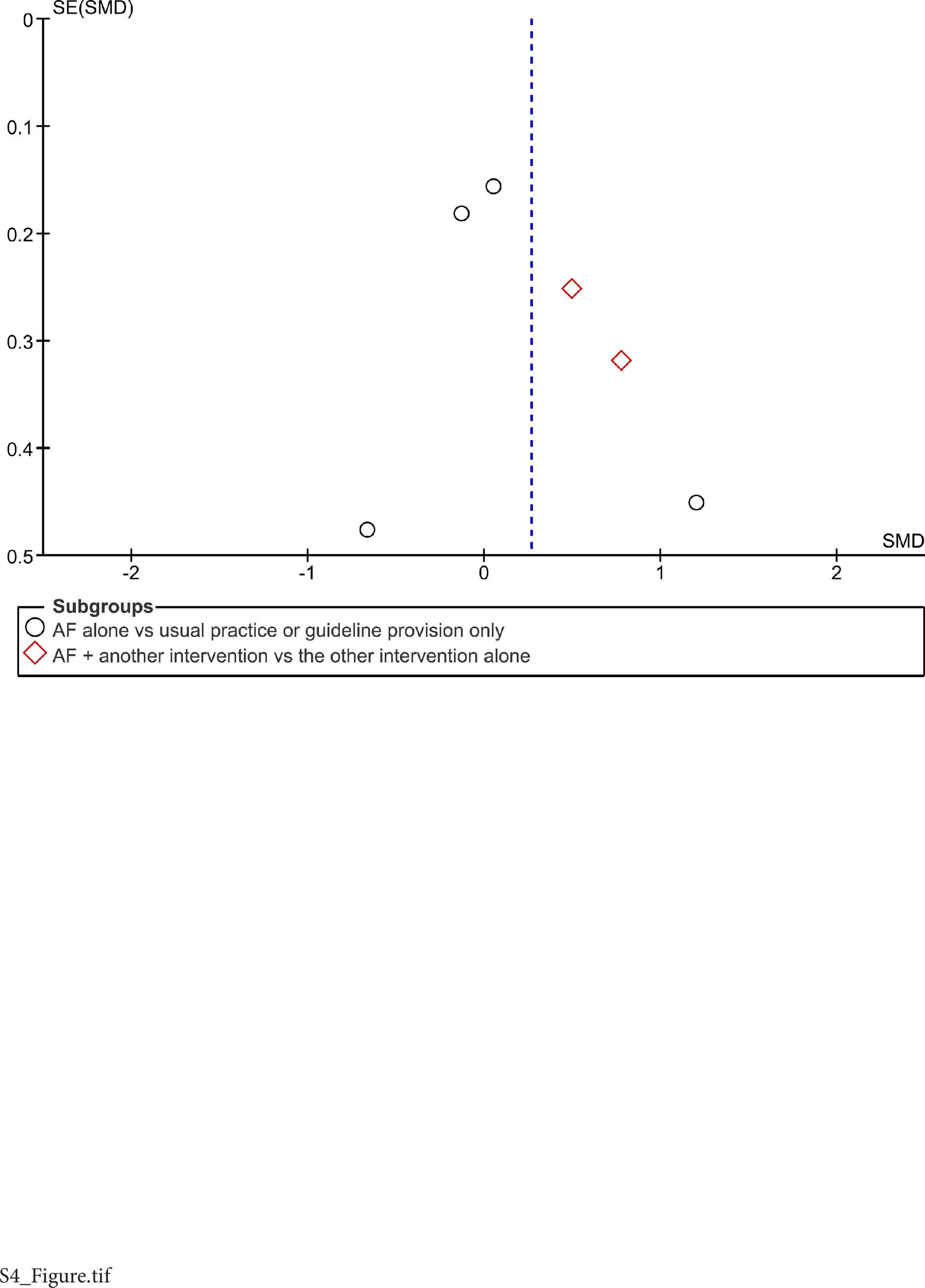

Supplement: S1 Appendix — S1 Fig. a. Effect of audit and feedback in observational studies on the number of diagnostic imaging requests (continuous outcome) (4–6). b. Effect of audit and feedback in observational studies on the number of diagnostic imaging requests (dichotomous outcome) (7, 8). S2 Fig. Effect of audit and feedback in observational studies on image order appropriateness (dichotomous outcome) (7). S3 Fig. Funnel plot of RCTs analyzing the total image order outcome. We did not consider this figure to be indicative of publication bias. The study in the bottom right favored the control intervention, not AF. S4 Fig. Funnel plot of RCTS analyzing the appropriateness of image orders outcome.We did not consider this figure to be indicative of publication bias. S1 Table. Description of AF interventions using TiDIER recommendations (1). Abbreviations: AF, Audit and Feedback; CT, Computed Tomography; Echo, Echocardiography; GIM, General physicians; Res, residents; Gov., Government; Mm; MRI, Magnetic Resonance Imaging; N/A, not applicable; PCP, Primary care physicians (e) PCPs refers to primary care physicians and may include family, general practice and general internal medicine physicians, (f) The term residents also refers to registrars (g) Comparison provided Includes own/ peers’ previous performance, national benchmark. Note: For multifaceted interventions, we assessed the characteristics of the audit and feedback component. S2 Table. a. Risk of Bias for NRCTs using the Risk Of Bias In Non-randomized Studies—of Interventions (ROBINS-I) tool (2). b. Risk of Bias for observational studies using Effective Practice and Organisation of Care (EPOC) recommendations (3). c. Risk of Bias for interrupted time series studies using Effective Practice and Organisation of Care (EPOC) recommendations (3). Legend: ● Low risk; ● Indeterminate Risk; ● High risk. S3 Table. Effect of audit and feedback in a non-randomized, crossover design study on the number of diagnostic imaging request 9).*no p-valu [file pone.0300001.s001.zip › S4_Fig.tif]
