# Supplementary material for: Audit and feedback to change diagnostic image ordering practices: A systematic review and meta-analysis
Source: PLoS One. 2024 Jun 5;19(6):e0300001. doi: 10.1371/journal.pone.0300001 (PMC11152319; doi:10.1371/journal.pone.0300001)
Supplement: Attachment — Submitted filename: PONE-D-23-34965_Review_CH.docx [file pone.0300001.s003.docx]

PONE-D-23-34965

Audit and Feedback to change diagnostic image ordering practices: A systematic review and meta-analysis

**Comments to editor**

Thank you for the opportunity to review this manuscript.

**Comments to author**

I thank the authors for providing the opportunity to review this manuscript. The authors aimed to assess the effect of auditing and feedback interventions directed at healthcare workers to reduce imaging orders. The authors ran a comprehensive search and included 11 RCT’s with 4311 participants in total. This review provides an interesting insight into the effects of audit and feedback interventions on the ordering of images in primary care, especially given the high rates of unnecessary imaging currently. There are some matters that will need to be addressed before this review could be considered suitable for publication.

Primarily, the concerns are regarding the way in which the studies have been pooled together. Different conditions will have a lower or higher threshold for further testing and pooling these studies together may not appropriate. For example, studies done on cardiologist will yield very different results to studies in a musculoskeletal setting as one condition (chest pain) would be considered life threatening compared to the other (i.e., knee pain)

**Major issues/comments**

**OVERALL**

- My impression is that the authors have looked at the effect of AF intervention to reduce imaging. My concern is that the pooling of studies with different conditions (i.e., musculoskeletal based studies vs cardiovascular studies) includes too much heterogeneity and means the results are not entirely interpretable. This is because the threshold for serious pathology will be different e.g., chest pain will likely get imaged and have a low threshold for further testing, whereas knee pain may have a higher threshold for further testing as it may not be considered life threatening.

**ABSTRACT**

**METHODS**

- One overall comment is that it may not be appropriate to pool studies that used diagnostic imaging for different body parts. For example, the threshold for suspicion of serious pathology is going to be different for the lumbar spine verses a pulmonary condition. The setting of which the imaging is ordered is also something that should be pooled separately. Serious pathology is more prevalent in emergency departments settings and clinicians may have a lower threshold to image compared to primary care. Therefore, pooling all the studies together does not seem appropriate due to the heterogeneity between studies. My suggestion would be pool studies based on musculoskeletal conditions, respiratory, cardiovascular etc. Happy for this to be discussed with the Editor, however a meta-analysis inclusive of all body parts seems inappropriate in my view.

**RESULTS**

**Study Characteristics**

- No description of participant characteristics e.g., age, gender

**Overall comment**

- This section is written well on a complicated issue. However as per the comments in the methods section, I do not feel that the way the data is presented accurately highlights the impact of AF interventions, as the effect will differ for different body parts. This is evident for example in Figure 2A Eccles 2001 (musculoskeletal) and Bhatia 2014 (cardiology) and the varying results of these two studies.

**DISCUSSION**

**Strengths and limitations**

- If the Editor decides the current meta-analysis is ok, then I think the heterogeneity between the studies need to be clearly stated. It needs to be clear that the pooling of different conditions adds to the heterogeneity in the data and means the interpretation of these results should be taken very cautiously. Again, my recommendation is to reconsider the methods.
