# Supplementary material for: Audit and feedback to change diagnostic image ordering practices: A systematic review and meta-analysis
Source: PLoS One. 2024 Jun 5;19(6):e0300001. doi: 10.1371/journal.pone.0300001 (PMC11152319; doi:10.1371/journal.pone.0300001)
Supplement: Attachment — Submitted filename: PONE-D-23-34965 Reviewer Comments.docx [file pone.0300001.s004.docx]

**Review comments**

Thank you for the opportunity to review this manuscript titled *“Audit and Feedback to change diagnostic image ordering practices: A systematic review and meta-analysis.”.* This is an important topic and will be of interest to administrators and policy makers looking to reduce overuse of imaging which wastes resources and can worsen patient outcomes. This is a high quality systematic review that follows EPOC recommendations, uses the TIDieR statement describe interventions and was prospectively registered.

**Abstract:**

Clear and concise, no suggestions for improvement.

**Introduction:**

Well written, clear, and relevant.

Line 5 – I think it would be more powerful to mention the types of unnecessary treatments, e.g. surgery and opioid use in the context of back pain/ The treatment cascade can have significant risk of harm, particularly if it arose from unnecessary testing. This could be emphasised.

Line 12 – Could you add the GRADE rating/certainty of evidence for the Cochrane review here?

**Methods:**

Lines 84 to 87 – Could you please expand further on how you judged appropriateness, e.g. which guidelines you compared the practice to, to decide if it was appropriate. Or, did the review authors reply on what the trial authors deemed appropriate? This could be more clear

Line 109 – Please specify if you used the Cochrane ROB tool 1 or 2

Lines 110 to 112 – Significant differences in base characteristics would be by nature due to random chance, assuming randomisation procedure was appropriate. Issues with randomisation would be picked up with the Cochrane ROB tool, therefore I don’t think this extra step is necessary.

Line 115 – In similar reviews, lack of a pre-published protocol is considered a risk for selective reporting. Could you please elaborate on why you did not consider this to be a risk?

**Results:**

Line 212 – Given the studies ranges from very low-moderate quality, consider clarifying that it is a low to moderate certainty evidence that AF can reduce imaging.

Line 233 – word “to” not required

**Discussion:**

Line 280-287 - Another implication that could be considered is how AF may reduce imaging for those who are typically underserviced, increasing harm to these patients. For instance, interventions and recommendations to reduce antibiotics for all children with otitis media can harm Indigenous children who are underserviced and under treated. Are there certain populations that could experience fewer DI than is clinically indicated?

Line 291 – It is interesting that “rarely appropriate” imaging requests had significant effects. You have provided the explanation of frequency, but could there also be clinician agreement and endorsement of the inappropriateness of imaging in those cases which increased the effect?

Line 315 – Other reasons a supervisor of colleague may be used is because this can threaten professional status and peer opinion. Clinicians may be motivated by wanting to maintain their reputation.

**Conclusion:**

Clear, includes statements about the certainty of evidence, sensible conclusion from the reported results.

**Tables and Figures:**

Figure 1 and 2 have AF on the left, but figure 3 has AF on the right. At a glance, this looks like the effects were in the opposite direction. If possible, keep consistent across figures.

Otherwise tables and figures are excellent.

**General**

Grammar and language and excellent throughout.
